# Circulating Neuronatin Levels Are Positively Associated with BMI and Body Fat Mass but Not with Psychological Parameters

**DOI:** 10.3390/nu15163657

**Published:** 2023-08-20

**Authors:** Amelie Rudolph, Andreas Stengel, Maria Suhs, Selina Schaper, Ellen Wölk, Matthias Rose, Tobias Hofmann

**Affiliations:** 1 Center for Internal Medicine and Dermatology, Department of Psychosomatic Medicine, Charité—Universitätsmedizin Berlin, Corporate Member of Freie Universität Berlin and Humboldt-Universität zu Berlin, 12203 Berlin, Germany; andreas.stengel@med.uni-tuebingen.de (A.S.);; 2 Department of Psychosomatic Medicine and Psychotherapy, University Hospital Tübingen, 72076 Tübingen, Germany; 3 Quantitative Health Sciences, Outcomes Measurement Science, University of Massachusetts Medical School, Worcester, MA 01655, USA; 4 Department of Psychosomatic Medicine, DRK Kliniken Berlin Wiegmann Klinik, 14050 Berlin, Germany

**Keywords:** gut-brain axis, anorexia nervosa, obesity, body composition, patient-reported outcome, psychosomatic

## Abstract

Human genetic studies have associated Neuronatin gene variants with anorexia nervosa (AN) and obesity. Studies on the expression of the Neuronatin gene product, a proteolipid, are lacking. We investigated the relationship between circulating Neuronatin, body mass index (BMI), body composition (BC), physical activity (PA), and psychometric outcomes in patients with AN, normal weight, and obesity. Plasma Neuronatin was measured by ELISA in (1) 79 subjects of five BMI categories (AN/BMI < 17.5 kg/m^2^; normal weight/BMI 18.5–25 kg/m^2^; obesity/BMI 30–40 kg/m^2^; obesity/BMI 40–50 kg/m^2^; obesity/BMI > 50 kg/m^2^) with assessment of BC (bioimpedance analysis; BIA); (2) 49 women with AN (BMI 14.5 ± 1.8 kg/m^2^) with measurements of BC (BIA) and PA (accelerometry); (3) 79 women with obesity (BMI 48.8 ± 7.8 kg/m^2^) with measurements of anxiety (GAD-7), stress (PSQ-20), depression (PHQ-9) and eating behavior (EDI-2). Overall, a positive correlation was found between Neuronatin and BMI (*p* = 0.006) as well as total fat mass (FM; *p* = 0.036). In AN, Neuronatin did not correlate with BMI, FM, or PA (*p* > 0.05); no correlations were found between Neuronatin and psychometric outcomes in obesity (*p* > 0.05). The findings suggest an FM-dependent peripheral Neuronatin expression. The decreased Neuronatin expression in AN provides evidence that Neuronatin is implicated in the pathogenesis of eating disorders.

## 1. Introduction

Genomic imprinting, a process of epigenetic modification resulting in a monoallelic expression of a subset of mammalian genes, has a critical role in fetal growth and development [1]. Not only prenatal but also postnatal viability is influenced by imprinted genes. Indeed, many of the approximately 200 genes identified have been shown to play key roles in metabolism [2]. This is underscored by the fact that several human genetic disorders characterized by altered expression of imprinted genes, for instance, Prader-Willi and Angelman syndrome, have been linked to metabolic diseases such as obesity and diabetes mellitus [3]. Recent data suggest that imprinted genes contribute to a wide range of adult behavior, especially social and feeding behavior [2]. Dysregulation of the genomic imprinting process is hypothesized to increase vulnerability to neuropsychiatric disorders [4].

Neuronatin (Nnat) is an imprinted gene that is transcribed exclusively from the paternal allele [5,6,7]. While the exact function of Nnat has not been fully elucidated to date, there is evidence for a role in the regulation of body weight since recent studies have associated single nucleotide polymorphisms in the human Nnat locus with extreme obesity on one [8] and with anorexia nervosa on the other hand [9].

The Nnat gene resides on human chromosome 20q within a large intron sequence of the adjacent bladder cancer-associated protein (BLCAP) gene [5]. By differential splicing, the Nnat gene encodes two distinct isoforms, in which solely the middle of three domains differs: Nnatα contains 81 amino acids and Nnatβ of 54 amino acids. Both isoforms include both a hydrophobic and a hydrophilic domain, resulting in sequence homology to proteolipids, and are hypothesized to regulate intracellular signaling via the endoplasmic reticulum calcium ATPase [10].

Nnat was first discovered in the developing brain during the embryonic period, where it induces neuronal differentiation by increasing intracellular Ca^2+^ levels [11,12]. Subsequent studies have shown that neuronal expression of Nnat is maintained throughout adulthood in several hypothalamic nuclei, including the arcuate nucleus (Arc), paraventricular nucleus (PVN), dorsomedial hypothalamic nucleus (DMH), and lateral hypothalamic area (LHA), in which Nnat colocalizes with various functional mediators of appetite regulation, for instance, cocaine- and amphetamine-regulated transcript (CART) and melanin-concentrating hormone (MCH) [8]. In the mouse hypothalamus, Nnat expression is dynamically regulated by metabolic status [8,13]. While fasting reduced the expression of Nnat in the Arc, PVN, and DMH [8,14], peripheral administration of the satiety hormone leptin resulted in an increased hypothalamic Nnat expression in the DMH and PVN [8].

Moreover, Nnat mRNA is abundantly expressed in peripheral tissues such as lungs, adrenal glands [15], stomach and jejunum [14], pancreas [16], and adipocytes in both white and brown adipose tissue [17]. At these various sites, pleiotropic functions related to energy homeostasis are discussed: In pancreatic β-cells, Nnat is required for the procession of preproinsulin and thus involved in glucose-stimulated insulin secretion [18]. Nnat is implicated in adipose tissue metabolism, as in vitro transfection of preadipocytes with Nnat-encoding vectors stimulates adipogenesis [17,19]. In white adipose tissue of obese mouse models, Nnat mRNA expression is increased [19,20], whereas bioinformatical analysis of subcutaneous human tissue showed a decrease in Nnat mRNA expression in obese subjects [21]. Indicative of the role of Nnat in regulating body weight, animal studies observed decreased physical activity and energy expenditure, hyperphagia, and a tendency towards obesity [22] in Nnat-deficient mice, although this finding has not been confirmed by all studies [23]. It has repeatedly been shown that murine Nnat deficiency results in an unusual bimodal weight distribution [24,25].

Taken together, evidence from animal and genetic studies points towards a role for Nnat in the regulation of energy balance and body weight. However, the physiological function of Nnat remains unresolved, and because human Nnat has only been studied in the context of gene expression analyses of adipose and brain tissue, the role of Nnat expression in human metabolism remains to be determined. In an attempt to approach human Nnat expression, we exploratively studied Nnat protein in the peripheral circulation from three distinct patient samples: To gain a better understanding of the putative role of Nnat in metabolic phenotypes, we (I) examined the relationship between circulating Nnat and body composition in a sample of patients across a broad body mass index (BMI) spectrum. Considering that Nnat is an imprinted gene, variations in the Nnat expression might be linked with behavioral patterns or neuropsychiatric endophenotypes. We, therefore (II), analyzed the association between circulating Nnat and psychometrically assessed anxiety, depressiveness, and eating disorder pathology in a group of female patients with obesity. Further, Nnat gene variants have been associated with an increased susceptibility for anorexia nervosa (AN) [9,26]. To investigate whether Nnat is differentially expressed in patients with AN, we (III) examined circulating Nnat and possible correlations with measures of physical activity and energy expenditure in female patients with AN.

## 2. Materials and Methods

### 2.1. Study Design and Participants

From 2010 to 2014, blood samples from patients at the Department of Psychosomatic Medicine, Charité—Universitätsmedizin Berlin, Germany, were collected to create a medical database that enables investigation of the relationships between endocrine regulatory circuits, body composition, and psychological processes. Applying an explorative and naturalistic approach, we recruited all patients diagnosed with typical or atypical AN according to ICD-10 (International Statistical Classification of Diseases and Related Health Problems of the World Health Organization (WHO), 10th revision) patients with obesity admitted for evaluation and initiation of treatment for weight loss or management of associated comorbidities, and normal-weight patients with somatoform or adjustment disorders. Exclusion criteria encompassed malignant diseases, somatoform or somatic disorders of the gastrointestinal system, hypercortisolism, current pregnancy, minority (age < 18 years), treatment with immunomodulatory drugs (e.g., methotrexate and azathioprine) or gastrointestinal surgery, especially bariatric surgery, with the exception of cholecystectomy.

The subjects were recruited upon patient admission in the Department of Psychosomatic Medicine at Charité- Universitätsmedizin Berlin and all of them gave written informed consent. The study was approved by the international ethics committee of the Charité- Universitätsmedizin Berlin (protocol number EA1/130/16).

In this study, three samples were extracted from the database to investigate the study’s objectives. 

#### 2.1.1. Sample 1: Patients with a Wide BMI Spectrum

For the first sample, a total of 79 patients representing five different BMI categories (BMI < 17.5 kg/m^2^/AN; BMI 18.5–25 kg/m^2^/normal weight (NW); BMI 30–40 kg/m^2^; BMI 40–50 kg/m^2^; BMI > 50 kg/m^2^) were selected from the database. The sample includes 42 female and 39 male subjects.

#### 2.1.2. Sample 2: Female Patients with Anorexia Nervosa

The second sample comprises 49 female patients admitted for treatment of diagnosed AN. Inclusion criteria encompassed fulfillment of ICD-10 criteria for typical or atypical AN and female sex. Seven subjects of this sample are also enrolled in the AN category of sample 1.

#### 2.1.3. Sample 3: Female Patients with Obesity

The third sample includes 79 female patients with obesity hospitalized for obesity-related somatic and mental comorbidities. Inclusion criteria for the OB group included a BMI > 30 kg/m^2^ and female sex. 13 subjects of this sample are also enrolled in sample 1 (Seven in BMI category 30–40 kg/m^2,^; four in BMI category 40–50 kg/m^2^; two in BMI category > 50 kg/m^2^).

### 2.2. Laboratory Analysis

Venous blood was drawn from all study participants within four days of admission and after an overnight fasting period. Patients were advised not to drink coffee, smoke, or exercise prior to blood withdrawal. Blood was sampled in pre-cooled EDTA tubes prepared with aprotinin (1.2 trypsin inhibitory units per 1 mL of blood; ICN Pharmaceuticals, Costa Mesa, CA, USA) for peptidase inhibition. After blood sampling, the tubes were immediately placed back on the ice and then centrifuged at 3000× *g* and 4 °C for 10 min. Plasma was then isolated and stored at −80 °C until further processing. Once the number of samples to be assayed was reached, plasma Nnat levels were measured using a commercial enzymatic immunoassay kit (ELISA, catalog # CSB-EL015905HU; Cusabio Biotech Co., LTD, Suffolk, UK). All samples were processed in one batch in February 2015; the intra-assay variability was <8% and the inter-assay variability was <10%.

### 2.3. Anthropometric Measurements

On the day of blood withdrawal, body weight and height were assessed. Measurements of weight were taken to the nearest 0.1 kg and height to the nearest 0.5 cm. Based on the results, BMI was calculated in kg/m^2^. Additionally, on the same day, the patient’s current medication and the presence of any exclusion criteria were recorded.

### 2.4. Assessment of Physical Activity and Energy Expenditure

PA and energy expenditure data were measured for three consecutive days starting on the day of blood withdrawal by using a portable armband device (SWA, SenseWear™ PRO3, BodyMedia Inc., Pittsburgh, PA, USA). Participants were not restricted to PA by the medical staff. The data were accepted and included in the analysis if patients wore the SWA for more than 20.5 h on at least two out of three days. Based on the measured activity and anthropometric data of the subjects, the SWA´s proprietary algorithm directly calculates the total amount of steps, the total energy expenditure (TEE), metabolic equivalents of tasks per day (MET), and the exercise-related activity thermogenesis (EAT, intentional physical activity, sport). Based on previous findings of Ainsworth et al. [27], energy expenditure of more than three metabolic equivalents of task (MET) was classified as EAT, whereas energy expenditure of up to three MET was classified as NEAT. The TEE value is composed of three components: resting energy expenditure (REE), the thermal effect of food (TEF), and activity thermogenesis, which can be further subdivided into exercise-related activity thermogenesis (EAT) and non-exercise activity thermogenesis (NEAT, spontaneous daily physical activity). As the SWA only calculates TEE and EAT, NEAT was calculated according to the equation NEAT = TEE − TEF − REE − EAT. The TEF required for the equation was estimated to be 10% of the TEE and thus calculated as TEE × 0.1, and the REE was approximated using the weight-group-specific REE prediction equations as described before [28].

### 2.5. Assessment of Body Composition

Body composition was measured by bioelectric impedance analysis (BIA) between 10:30 A.M. and 01:00 P.M. on the day of blood sampling under standardized conditions. Subjects were required to fast for at least two hours and remain in the supine position for at least half an hour prior to BIA measurements. Using equations provided by the manufacturer of the bioelectric impedance analyzer (NutriPlus, Data Input), phase angle, fat mass, fat-free mass (FFM), body cell mass (BCM), and extracellular mass (ECM) of the subjects were calculated.

### 2.6. Patient-Reported Outcomes

For patient-reported outcomes on psychological parameters, all subjects of the third sample (OB group) were asked to complete four different self-assessment questionnaires. Subjects were excluded if psychometric data were not obtained within 5 days of blood sampling.

The German version of a nine-item subscale of the Patient Health Questionnaire (PHQ) [29], a reliable and well-validated screening tool for major depression, was applied for the assessment of depressive symptoms. A score ranging from 0 to 27 determines the severity of depression, and at a cut-off value of ≥10, the sensitivity and specificity of the PHQ-9 for the detection of depression are 0.85, respectively [30].

To examine symptoms of generalized and social anxiety, panic disorders, and posttraumatic stress, we used the German version [31] of the Generalized Anxiety Disorder Questionnaire (GAD) [32], a subscale of the PHQ. It consists of seven items with scores ranging from 0 (not at all) to 3 (nearly every day), with a maximum of 27 points. Spitzer, Kroenke, Williams, and Löwe [31] found that the GAD-7 score is an excellent measure of anxiety symptom severity, with cut-off values of 5, 10, and 15 representing mild, moderate, and severe anxiety, respectively.

The German version [33] of the perceived stress questionnaire (PSQ) [34] with 20 items was used to measure stress as perceived by the individual. It encompasses four subscales: “worries”, “tension”, and “joy” as stress reactions and “demands” as perceptions of external stressors. 

Disordered eating symptoms were determined with the German version [35] of the short form of the second version of the Eating Disorder Inventory (EDI) [36]. EDI-2 contains 64 items on 8 subscales assessing the ‘drive for thinness’, ‘bulimia’, ‘body dissatisfaction’, ‘ineffectiveness’, ‘perfectionism’, ‘interpersonal distrust’, ‘interoceptive awareness’, and ‘maturity fears’. The obtained values were extrapolated to scores ranging from 0 to 100. 

### 2.7. Statistical Analysis

Data are expressed as mean ± standard deviation (SD). The distribution of the data was evaluated using the Kolmogorov–Smirnov test. The correlation was assessed by Spearman’s analysis. To examine differences between the groups, between-group comparisons were performed using either the Kruskal–Wallis test followed by the Dunn–Bonferroni post-hoc test for nonparametric data or the chi-square test for parametric data. Differences were considered significant if *p* < 0.05. To identify outliers, we set a cut-off of three SD from the mean Nnat value of each group. Two outliers in sample 1 (one in the AN group, one in the obesity BMI 40–50 group) were detected and excluded from further statistical analysis. Multivariable linear regression analysis was employed to investigate the effect of possible confounders, such as comorbidities and medications, on Nnat.

## 3. Results

### 3.1. General Characteristics of the Study Samples

#### 3.1.1. Sample 1

General characteristics, medication, and bioelectrical impedance analysis parameters of subjects in Sample 1 are presented in Table 1. The study groups did not differ in age except for the subjects with AN who were younger (*p* < 0.01, Table 1). As anticipated, BMI differed significantly between all study groups, with the lowest value in the AN group (*p* < 0.05, Table 1). For body composition calculated using BIA, the AN and NW groups exhibited significantly lower fat mass, lean mass, total body water, FFM, BCM, and ECM than all three OB groups (*p* < 0.05, Table 1). AN and NW groups differed significantly only in terms of total body water, FFM, and BCM (*p* < 0.05, Table 1), each with lower values in the AN group. Furthermore, as expected, there was a significantly lower number of subjects with type 2 diabetes mellitus, arterial hypertension, hypertriglyceridemia, and fatty liver disease in the AN and normal weight groups compared to the three OB groups (*p* < 0.05, Table 1). Psychopharmacological treatment was received by 24% of the sample population, with SSRI/SSNRI (8%), tricyclic antidepressants (6%), and other antidepressants (6%) being the most common. There were no significant differences in medication prescription between the groups except for the higher steroid intake in the BMI 30–40 group (*p* < 0.05, Table 1).

Due to the much higher prevalence of anorexia nervosa in women, the group of subjects with anorexia nervosa consisted only of female patients. Therefore, for the study of sex differences in Nnat concentration and age, the AN group was excluded. No differences between male and female subjects were observed in Plasma Nnat (34.5 ± 24.9 vs. 27.2 ± 21.6 pg/mL, *p* = 0.224). Moreover, male and female subjects did not differ in age (42.2 ± 12.9 vs. 44.2 ± 13 years, *p* = 0.535) or BMI (42.8 ± 16.7 vs. 41 ± 17.5 kg/m^2^, *p* = 0.657).

#### 3.1.2. Sample 2

General characteristics, body composition parameters, and activity parameters of study Sample 2, female subjects with anorexia nervosa, are presented in Table 2. Data on activity parameters were missing for four subjects, leaving data from 45 subjects available for analysis. There was a broad range in activity levels across the subjects, indicated by the number of steps (range 2087–37,750, mean ± SD: 10,748 ± 6335, Table 2) and duration of exercise (0–107 min/d, mean ± SD 15.8 ± 21.9 min/d, Table 2). Comorbidities and medical characteristics of the sample are presented in Table 3.

#### 3.1.3. Sample 3

General characteristics and psychometrical data of Sample 3, female subjects with obesity, are shown in Table 4. The mean BMI of the sample was 48.8 ± 7.6 kg/m^2^, thus reflecting severe obesity as defined by WHO (BMI cut-off > 40 kg/m^2^). The age of the subjects ranged from 19–73 years, with a mean age of 44.8 ± 13.7 years. For the psychometric assessments, PHQ data were missing for one subject, and EDI data were unavailable for four subjects. The sample covered a wide range of psychopathology: The scores for depressiveness (PHQ) ranged from 0–25, with a mean of 9.9 ± 6.1, indicating moderate depressiveness (10–15) on average [29]. With a range of 0–21 and a mean of 9.2 in anxiety scores (GAD-7), the average subject displayed mild anxiety symptoms (scores of 5, 10, and 15 are considered cut-offs for mild, moderate, and severe anxiety, respectively [31]). As reflected by the range of the scores, the sample also showed a broad spectrum of eating disorder symptoms in EDI (range 11–79, mean 45.9 ± 14) and perceived stress levels in PSQ (range 5–98, mean 55.4). Comorbidities and medical characteristics of the sample are presented in Table 3.

### 3.2. Circulating Nnat Is Positively Correlated with BMI and Body Fat Mass in Patients over a Wide BMI Spectrum

**Figure 1:**
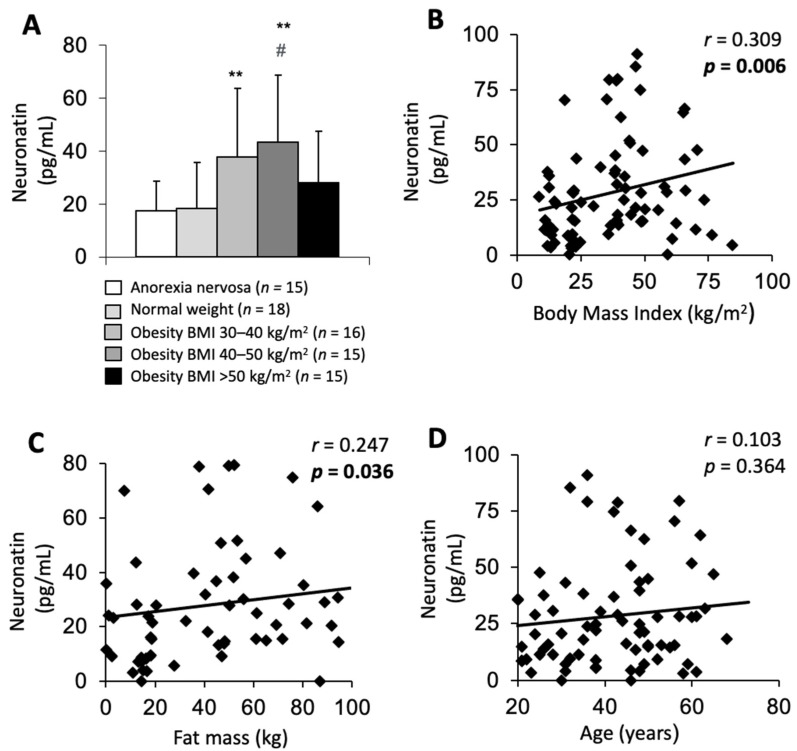
In Sample 1 (*n* = 79), obese subjects in group BMI 40–50 displayed significantly elevated plasma Nnat levels compared to subjects in AN and normal weight group, and obese subjects in group 30–40 kg/m^2^ had higher plasma Nnat levels than subjects in normal weight group (**A**). Over the whole sample, plasma Nnat is positively correlated with body mass index in kg/m^2^ (**B**) and fat mass in kg (**C**) but not with age in years (**D**). Data are expressed as mean, standard deviation. Values for *r* and *p* are indicated in each correlation graph. ** *p* < 0.01 vs. Anorexia nervosa group, # *p* < 0.05 vs. Normal weight group.

The mean Plasma Nnat in Sample 1 was 28.8 ± 22.5 pg/mL (range: 3.1–90.8 pg/mL). We observed a weak positive correlation between Nnat and BMI in the whole of Sample 1 (r = 0.309, *p* = 0.006; Figure 1B). Obese subjects in BMI groups 30–40 and 40–50 exhibited significantly higher Nnat levels than normal weight subjects (*p* = 0.006 NW vs. BMI 30–40, *p* = 0.001 NW vs. BMI 40–50, Table 1, Figure 1A), and Nnat levels in BMI group 40–50 were significantly higher than in the AN group (*p* = 0.002, Table 1, Figure 1A). Subjects in the BMI group > 50 showed comparable Nnat levels to subjects in NW and AN groups (*p* > 0.05). Over the whole sample, fat mass as calculated by BIA measurement correlated positively with Nnat levels, either expressed in kg (r = 0.247, *p* = 0.036; Figure 1C) or as % of body weight (r = 0.268; *p* = 0.023). No other body measurement parameter was associated with Nnat, although the required significance level was only marginally missed (FFM r = 0.229, *p* = 0.053; ECM r = 0.230, *p* = 0.052; BCM r = 0.221, *p* = 0.062; Body water r = 0.229, *p* = 0.053). Furthermore, age did not correlate with Nnat (r = 0.103, *p* = 0.364; Figure 1D).

A stepwise multiple linear regression analysis examining medications as possible confounders showed that BMI predicted Nnat levels (*p* = 0.044), while insulin (*p* = 0.222), steroids (*p* = 0.251), DPP-4 antagonists/GLP-1 analogs (*p* = 0.728), psychopharmacological treatment (*p* = 0.249), antipsychotics (*p* = 0.249), SSRI/SNRI (*p* = 0.187), tricyclic antidepressants (*p* = 0.176) and other antidepressants (*p* = 0.16) did not. However, in a stepwise multilinear regression analysis, the association between BMI and plasma Nnat levels was lost when comorbidities were included as possible confounders (*p* = 0.211), while the presence of fatty liver disease predicted plasma Nnat levels (*p* < 0.001). Other comorbidities such as sleep-related breathing disorders (*p* = 0.111), type 2 diabetes mellitus (*p* = 0.938), arterial hypertension (*p* = 0.240), hypercholesterolemia (*p* = 0.274), and binge eating disorder (*p* = 0.457) were not predictive of Nnat plasma levels.

### 3.3. Circulating Nnat Is Positively Correlated with Fat-Free Mass, Extracellular Mass, and Total Body Water but Not with Other Measures of Body Composition, Activity, or Energy Expenditure in AN Patients

**Figure 2:**
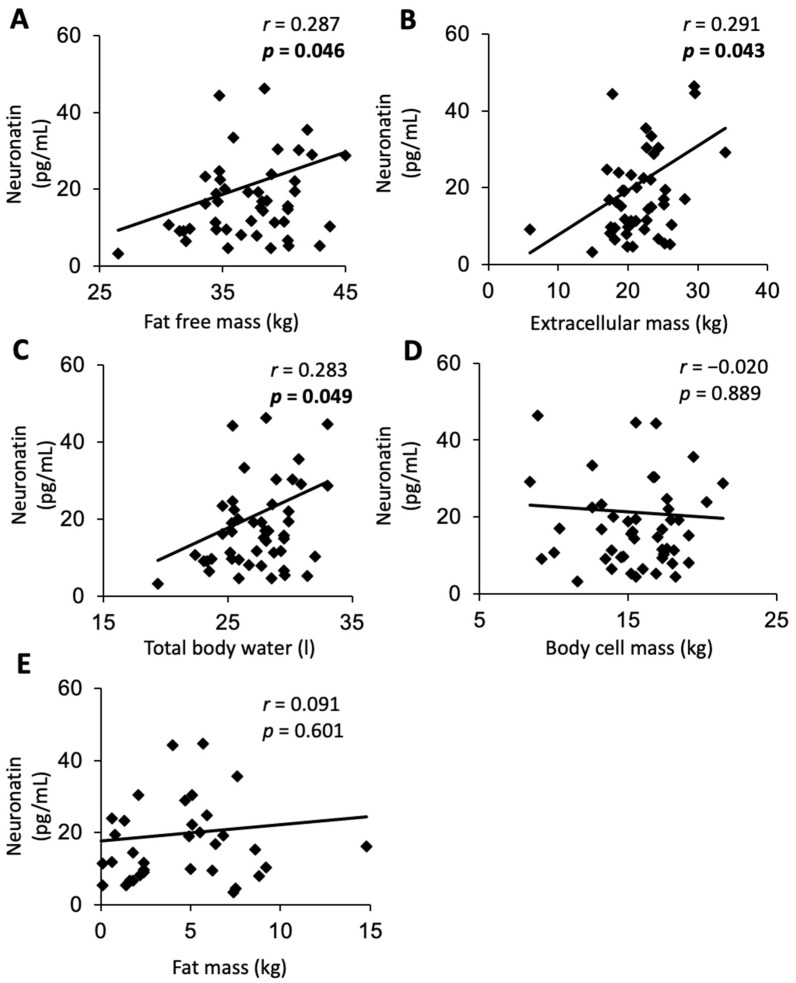
In Sample 2, females with anorexia nervosa (*n* = 49), plasma Nnat levels showed a positive correlation with fat-free mass (**A**), extracellular mass (**B**), and total body water (**C**), but not with other body composition parameters such as body cell mass (**D**) and fat mass (**E**). Values for r and *p* are indicated in each correlation graph.

In Sample 2, the mean plasma Nnat concentration was 20.8 ± 20.6 pg/mL (range: 3.3–109.7 pg/mL). Nnat did not show an association with BMI, age, and all measured activity parameters (*p* > 0.05). However, we observed weak positive correlations between peripheral Nnat levels and fat-free mass (r = 0.287, *p* = 0.046; Figure 2A), extracellular mass (r = 0.291, *p* = 0.043; Figure 2B), and total body water (r = 0.283, *p* = 0.049; Figure 2C). No correlation was found with other body composition parameters such as fat mass (r = −0.091, *p* = 0.601; Figure 2D) and body cell mass (r = 0.020, *p* = 0.889; Figure 2E).

### 3.4. Circulating Nnat Is Not Associated with Patient-Reported Psychological Outcomes in Obese Patients

Sample 3 exhibited a mean plasma Nnat of 37.1 ± 7.7 pg/mL and a range of 0.2–111.9 pg/mL. We did not observe correlations between Nnat and anxiety (GAD-7; *r* = −0.07, *p* = 0.539), depressiveness (PHQ-9; *r* = −0.083, *p* = 0.469), eating disorder pathology (EDI-2; *r* = −0.083, *p* = 0.481), and perceived stress (PSQ-20; *r* = 0, *p* = 0.999). In this sample, Nnat levels correlated weakly and positively with age (*r* = 0.282, *p* = 0.012) but not with BMI (r = 0.196, *p* = 0.084). In a multiple regression analysis, age was the strongest predictor of plasma Nnat levels (*p* = 0.006), and likewise, BMI showed a positive association with Nnat *(p* = 0.020), whereas scores in PHQ-9 (*p* = 0.668), GAD-7 (*p* = 0.539), PSQ-20 (*p* = 0.085) and EDI-2 (*p* = 0.280) were not predictive of Nnat.

## 4. Discussion

Here we present the first exploratory data on the distribution of circulating Nnat in humans. We analyzed anthropometric and psychometric parameters as well as activity and energy expenditure data in patient samples with different body weights and in relation to circulating Nnat. We detected Nnat in the plasma of all study subjects and observed a positive correlation between Nnat, BMI, and body fat in patients over a very wide BMI spectrum. Examining other possible characteristics of Nnat, we found no association with physical activity or energy expenditure in a sample of patients with AN and a broad range of steps completed per day and observed no relationships with depression, anxiety, stress, or eating disorder pathology in a sample of patients with severe obesity.

The positive correlation between Nnat, BMI, and body fat might indicate that Nnat is implicated in body weight regulating mechanisms, as suggested by a human genetic study that associated Nnat gene variants with severe childhood and adult obesity [8]. 

Studies in animals have pointed towards a role of Nnat in satiety pathways as neuronal Nnat has been shown to colocalize with various functional mediators of appetite regulation in the LHA [8], and Nnat-positive neurons in the solitary tract are activated by peripheral satiety signals such as cholecystokinin and bombesin [37]. In mice, it has been demonstrated that hypothalamic Nnat expression is downregulated during fasting and upregulated following leptin injection [14], and Nnat-deficient mice showed hyperphagia and susceptibility to obesity in old age [22]. Since a body weight-dependent expression pattern, downregulation under fasting conditions and induction of hyperphagia upon deprivation are known properties of anorexic peptides, one may hypothesize that Nnat also acts as an anorexic signal through various central and peripheral pathways.

However, when interpreting these results, it should be noted that the correlation we found with BMI was weak and not replicated in the samples of obese and anorexic females, most likely due to the smaller BMI range compared with the first sample. Moreover, as reported in 3.2, the presence of fatty liver disease was the strongest predictor of Nnat levels in a multiple regression analysis. Nonalcoholic fatty liver disease, also considered as hepatic manifestation of metabolic syndrome, is characterized by hepatic accumulation of lipids leading to lipotoxicity and oxidative stress, both of which result in chronic, low-grade liver inflammation that can eventually progress to the more severe manifestation, nonalcoholic steatohepatitis [38]. The exact number of cases of fatty liver disease in our study sample attributable to a nonalcoholic etiology cannot be specified, but it can be assumed that a substantial proportion of the patients studied developed fatty liver disease due to severe obesity rather than excessive alcohol consumption. However, chronic inflammation in individuals with fatty liver disease may provide an explanation for the observed association with circulating Nnat. Mzhavia et al. [39] demonstrated increased expression of Nnat in endothelial cells extracted from obese and diabetic mice, suggesting its involvement in chronic inflammatory processes commonly reported in the context of obesity and aging [40]. This explanatory approach is also applicable to the observation that age was the strongest predictor of Nnat (*p* = 0.006) and positively correlated with Nnat (*p* < 0.001) in obese females. Another factor worth considering is the higher prevalence of comorbidities and medications in older patients, which may alter the expression of proteins involved in body weight regulation. Nevertheless, the correlation between age and Nnat was not observed in samples 1 and 2, rendering it unstable. Overall, Nnat expression appears to be regulated by a variety of factors that require further investigation.

When interpreting the results, it should be taken into consideration that the circulating Nnat measured in this study is intrinsically dependent on the expression activity of the paternal imprinted Nnat gene, which is the subject of recent studies:

In genetic research, a long-standing issue is that monozygotic twins can exhibit metabolically distinct phenotypes that cannot be explained by genetic or environmental variables, suggesting an additional dimension that contributes to trait variation [41]. 

These unexplained phenotypic variations (UPV) may be mediated by residual genetic variations [42], mosaic genetic variations, or epigenetic processes such as gene-environment interactions, but the molecular machinery is still poorly understood and often considered as random biological “noise”. However, recent epigenetic data have shown that at least part of UPV can be explained by single gene loci that are regulated in a probabilistic manner and can generate a bimodal “on” or “off” state of gene expression [43].

Interestingly, a recent study by Yang et al. [44] pointed out that the Nnat gene acts as a buffer against UPV: In isogenic mice, deletion of Nnat led to the development of bi-stable polyphenism with two distinct phenotypic states referred to as “normal” and “overgrown”. Mice of the “overgrown” population showed increased lean and fat mass, as observed in previous studies in rodents with Nnat deficiency [22,24]. Consistent with these results, in a human monozygotic twin cohort, Yang et al. [44] identified two distinct phenotypic variation patterns: Type A and Type B UPV, of which the latter is characterized by increased fat and lean mass with concomitant reduced Nnat mRNA expression. In our sample of morbidly obese females, we discovered a wide range (0.2–119.9 pg/mL) of circulating Nnat, with a minimum of 0.2 pg/mL. Since decreased Nnat expression was associated with an increase in fat and lean mass in the aforementioned study by Yang et al., one could speculate that our sample 3 contained an indeterminate number of phenotypic subtypes resembling Type B UPV, exhibiting decreased Nnat expression and, consequently, intrinsic susceptibility to the development of obesity. It has been suggested that the genetic component affecting obesity is higher in individuals with particularly severe obesity, as family members of severely obese subjects were found to be eight times more likely to develop obesity than the general population [45]. 

Consistent with this, mean Nnat levels in subjects with a BMI of more than 50 kg/m^2^ (as defined by WHO: superobese individuals) were similarly low as in normal-weight and anorexic subjects. However, the observation of an overall positive correlation between circulating Nnat, BMI, and body fat rather contradicts this hypothesis. For a more detailed insight into Nnat expression patterns of obese individuals, interventional studies measuring peripheral Nnat after food consumption and longitudinal studies examining Nnat expression after induced weight gain or weight loss are necessary.

In sample 1 with patients over a wide BMI spectrum, subjects with AN showed Nnat levels that were almost threefold reduced compared to obese subjects. The finding is further corroborated by the observation that females with AN in sample 2 exhibited significantly lower mean Nnat levels than females with obesity in sample 3. The first possible explanation for this observation is the downregulation of Nnat expression in phases of extreme hunger. Consistent with this, preclinical studies demonstrated that both obese and lean rats fasted for 48 h exhibited a decrease in hypothalamic Nnat mRNA expression [14]. Another explanation, based more on genetic evidence, could be that variants of the Nnat gene with decreased Nnat expression result in lower body weight or susceptibility to developing an eating disorder. This hypothesis is consistent with the findings of Lombardi et al. [9], who linked single nucleotide polymorphisms (SNP) in the Nnat gene to increased susceptibility to AN. Subsequently, a recent in silico study [46] identified a Nnat SNP missense variant that strongly affects gene expression and is also hypothesized to interact with drugs commonly used in AN therapy, which could lead to implications for treatment efficacy. However, the extent to which the described variants lead to increased or decreased expression of Nnat gene products remains unclear.

Moreover, the molecular links between altered Nnat expression and AN pathophysiology are still elusive. Several studies in rodents showed that altered Nnat expression leads to changes in locomotor activity and energy expenditure, as mice with global Nnat deletion displayed increased activity profiles [24] and reduced energy expenditure [22]. However, in our sample of subjects with AN, we did not detect any correlations between circulating Nnat and physical activity, TEE, or other parameters of energy expenditure, especially EAT. Therefore, a possible implication of Nnat in the pathogenesis of AN is unlikely to be explained by triggering changes in activity. Another possible link between altered Nnat gene expression and susceptibility to the development of eating disorders might be that Nnat is implicated in psychological processes. We did not observe any correlation between Nnat and psychometrically assessed anxiety, depression, perceived stress, and eating behavior pathology. Considering that Nnat has never been analyzed in the context of psychological processes, neither in animals nor in humans, further research (e.g., of experimental design) is warranted in this field.

Moreover, we selected a sample of obese females to investigate the relationship between Nnat and psychometric parameters because mood and anxiety disorders are more prevalent in obese individuals [47], and the prevalence of anxiety disorders and pathological eating behaviors is even higher in females than in males [48]. However, it would be interesting to perform a similar measurement in patients with anorexia, as a modification of Nnat expression in the pathophysiology of AN seems conceivable, considering the current genetic research mentioned above.

The present study is subject to several limitations that must be taken into consideration:

First, we used a commercial ELISA Kit (# CSB-EL015905HU; Cusabio Biotech Co., LTD, Suffolk, UK) designed for measuring Nnat proteolipid in different human tissues and biological fluids. Although no significant cross-reactivity or interference between human Nnat and analogs was observed, the manufacturer stated that due to the limited current knowledge of Nnat, cross-reactivity with unknown other proteolipids may be feasible. In addition, two different isoforms of the proteolipid Nnat are known that may exert different functions, and the manufacturer did not specify which isoform could be detected by the ELISA antibodies. 

Second, it should be remembered that the current evidence of an association between Nnat and body weight dysregulation in humans is based on genetic studies. Although plasma Nnat proteolipid levels may reflect peripheral Nnat expression, our study obviously does not allow conclusions about central Nnat gene regulation. Further, references to the functions of Nnat originate exclusively from animal studies. Thus the transferability of the results to humans is unclear. 

Third, we analyzed body composition with BIA and activity parameters with SWA technology, both of which are well-established methods in clinical practice and research. However, BIA does not provide a definitive measurement of the various body components under conditions of highly altered BMI [49]. In addition, SWA may not be as accurate as the reference method of indirect calorimetry in evaluating subjects with AN, given that SWA has been shown to overestimate resting energy expenditure by 23% ± 27% in these subjects [50]. Moreover, although the subjects were not restricted by medical staff, their activity may not be representative of activity in the home setting. 

Lastly, we applied a naturalistic approach to the assessment of Nnat expression, which generally does not include a healthy control group. Our study samples exhibited heterogeneous comorbidities and diverse medications, which represent potential confounders for the results obtained and could contribute to the weak and rather unstable correlation between Nnat and BMI. This approach also implied that we did not select subjects to achieve a specific sample size but included every patient without exclusion criteria, resulting in our database containing an unequal number of subjects with AN and obesity.

However, the naturalistic study design, along with the inclusion of three different patient samples, is also a strength of the study, as our findings are more likely to be applicable to real-life settings and thus provide greater ecological validity.

## 5. Conclusions

In the present study, we demonstrated a positive correlation between circulating Nnat and BMI, suggesting an involvement of Nnat in the regulation of body weight, potentially as an anorexigenic agent. We found a reduction in peripheral Nnat expression in subjects with AN compared with subjects with obesity, consistent with the observation of genetic studies that have associated variations of Nnat gene expression with the pathophysiology of AN. Further investigations of both Nnat’s molecular modes and its peripheral and central expression in humans are warranted. Moreover, to fully elucidate the relevance of Nnat as a mediator or moderator of body weight, metabolic functions, and psychological processes, further extensive research is required, particularly interventional and longitudinal studies in more homogenous and larger study samples. For instance, a longitudinal study of patients with AN before and after hospitalization could determine whether decreased Nnat levels are attributable to an extremely low body fat mass at baseline or, if still significantly reduced after treatment, may point towards a Nnat gene variant with decreased protein expression and thus possibly increased susceptibility to AN.

## Figures and Tables

**Table 1:** General characteristics, Comorbidities, and Medication of Sample 1 (mixed body mass index categories).

Parameter	Group											
	Whole Sample	*n*	Normal Weight	*n*	Anorexia Nervosa	*n*	Obesity 30–40	*n*	Obesity 40–50	*n*	Obesity > 50	*n*
General Characteristics												
Sex (F/M)	42/37	79	9/9	18	15/0	15	7/9	16	6/ 9	15	5/10	15
Age(years)	39.5 ± 4.7	79	41.4 ± 15.2 ^§§^	18	26. 3± 9.6 ^###,+++,**^	15	43.9 ± 11.5	16	47 ± 12.4	15	40.7 ± 12.4	15
BMI (kg/m^2^)	36.8 ± 18.8	79	22.1 ± 1.5 ^§,#,+++,***^	18	12.7 ± 1.8 ^###,+++,***^	15	37.4 ± 2.7 ^***^	16	45.7± 3	15	65.6 ± 8.7	15
Plasma Neuronatin (pg/mL)	28.8 ± 22.5	79	18.5 ± 17.1 ^#,++^	18	17.5 ± 11.3 ^++^	15	37.9 ± 25.8	16	43.4 ± 25.4	15	28.1 ± 19.5	15
Bioelectrical impedance analysis												
Fat mass (kg)	46.5 ± 38.4	79	16 ± 4.5 ^#,+++,***^	18	−1.21 ±2.9 ^###,+++,***^	15	46.3 ± 7.8 ^***^	16	63.6 ± 12.7	15	104 ± 22.1	15
Fat mass (%)	32.9 ± 20.7	79	24.22 ± 6 ^##,+++,***^	18	−4.53 ± 2.9 ^###,+++,***^	15	40.6 ± 7.2 ^*^	16	46 ± 7.4	15	52.4 ± 7.5	15
Total body water (l)	48 ± 17.4	79	37 ± 7.1 ^§,#,+++,***^	18	25.5 ± 2.4 ^###,+++,***^	15	50.9 ± 10 ^*^	16	55.1 ± 10	15	69.9 ± 14	15
Fat-free mass (kg)	65.6 ± 23.8	79	50.6 ± 9.7 ^§,#,+++,***^	18	34.9 ± 3.3 ^###,+++,***^	15	69.6 ± 13.7 ^*^	16	75.3 ± 13.7	15	95.6 ± 19.1	15
Extracellular mass (kg)	33.4 ± 12.9	79	24.5 ± 4 ^##,+++,***^	18	20 ± 4.8 ^###,+++,***^	15	33.4 ± 6.1 ^**^	16	38.1 ± 7.4	15	50.5 ± 12.9	15
Body cell mass (kg)	32.7 ± 13.1	79	26.1 ± 6.3 ^§,#,+++,***^	18	13.9 ± 3 ^###,+++,***^	15	36.8 ± 8.5	16	37.2 ± 7.3	15	47.8 ± 10	15
Comorbidities												
Binge-Eating Disorder	8 (10%)	79	0 (0%)	18	0 (0%)	15	3 (15%)	16	4 (20%)	15	5 (25%)	15
Sleep-associated breathing disorder	38 (38%)	79	0 (0%)	18	0 (0%)	15	10 (50%)	16	13 (65%)	15	15 (75%)	15
Type 2 diabetes mellitus	16 (20%)	79	0 (0%) ^***^	18	0 (0%) ^***^	15	3 (29%)	16	4 (27%)	15	8 (53%)	15
Arterial hypertension	38 (47%)	79	3 (16%) ^#,+++,**^	18	1 (7%) ^###,+++,***^	15	10 (63%)	16	12 (80%)	15	12 (80%)	15
Hypercholesterinemia	49 (61%)	79	11 (61%)	18	6 (40%)	15	11(69%)	16	9 (60%)	15	12 (80%)	15
Hypertriglyceridemia	19 (24%)	79	0 (0%) ^##,*^	18	0 (0%) ^##,*^	15	8 (50%)	16	4 (27%)	15	6 (40%)	15
Fatty liver disease	32 (40%)	79	0 (0%) ^###,+++,***^	18	1 (7%) ^###,+++,***^	15	11 (71%)	16	10 (69%)	14	10 (71%)	14
Medication												
Insulin	5 (6%)	79	0 (0%)	18	0 (0%)	15	0 (5%)	16	3 (20%)	15	2 (13%)	15
DDP-4 antagonists/GLP-1 analogs	1 (1%)	79	0 (0%)	18	0 (0%)	15	0 (0%)	16	0 (0%)	15	1 (7%)	15
Antidiabetics	8 (10%)	79	0 (0%)	18	0 (0%)	15	3 (19%)	16	2 (13%)	15	3 (20%)	15
Steroids	7 (9%)	79	1 (6%) ^#^	18	0 (0%) ^#^	15	7 (31%)	16	0 (0%) ^#^	15	0 (0%) ^#^	15
Opioids	4 (5%)	79	0 (0%)	18	0 (0%)	15	1 (6%)	16	1 (7%)	15	2 (13%)	15
Psychopharmacological treatment	19 (24%)	79	5 (28%)	18	3 (20%)	15	4 (25%)	16	3 (20%)	15	4 (27%)	15
Antipsychotics	5 (6%)	79	0 (0%)	18	2 (13%)	15	1 (6%)	16	0 (0%)	15	2 (13%)	15
SSRI/SNRI	6 (8%)	79	0 (0%)	18	1 (7%)	15	2 (13%)	16	1 (7%)	15	2 (13%)	15
Tricyclic antidepressants	3 (4%)	79	1 (6%)	18	0 (0%)	15	1 (6%)	16	0 (0%)	15	1 (7%)	15
Other antidepressants	5 (6%)	79	2 (11%)	18	2 (13%)	15	0 (0%)	16	0 (0%)	15	1 (7%)	15
Tranquilizers, sedatives, hypnotics	2 (3%)	79	1 (6%)	18	0 (0%)	15	0 (0%)	16	1 (7%)	15	0 (0%)	15
Other psychopharmacological medication	6 (8%)	79	1 (6%)	18	0 (0%)	15	0 (0%)	16	2 (13%)	15	3 (20%)	15

Data are expressed as mean ± standard deviation. All group comparisons are calculated by Kruskal–Wallis and Dunn–Bonferroni Post-hoc tests. Abbreviations: BMI, body mass index; DPP-4, dipeptidyl peptidase-4 inhibitor; GLP-1, glucagon-like peptide-1; SSRI, selective serotonin reuptake inhibitors; SNRI, serotonin-norepinephrine reuptake inhibitors. Significant differences between the groups are displayed as: ^§§^ *p* < 0.01 ^§^ *p* < 0.05 (vs. Anorexia nervosa); ^###^ *p* < 0.001, ^##^ *p* < 0.01, ^#^ *p* < 0.05 (vs. Obesity BMI 30–40) ^+++^ *p* < 0.001, ^++^ *p* < 0.01(vs. Obesity 40–50); ^***^ *p* < 0.001, ^**^ *p* < 0.01, ^*^ *p* < 0.05 (vs. Obesity > 50).

**Table 2:** General characteristics and activity parameters of Sample 2 (female patients with anorexia nervosa, *n* = 49).

General Parameter	Mean ± SD	Range	*n*
Plasma Nnat (pg/mL)	21.3 ± 20.7	3.3–109.7	49
Age (years)	27.8 ± 9.4	18–52	49
BMI (kg/m^2^)	14.5 ± 1.8	10.5–18	49
Activity parameter			45
Number of steps/d	10,748 ± 6335	2087–37,750	45
Metabolic equivalents/d	1.8 ± 0.3	1.4–2.7	45
Total energy expenditure (kcal/kg/d)	43.9 ± 6	133.5–61.1	45
Resting energy expenditure (kcal/kg/d)	17.8 ± 1.2	14.8–19.6	45
Duration of exercise (min/d)	15.8 ± 21.9	0–107	45
Exercise activity thermogenesis (kcal/kg/d)	1.5 ± 2.2	0–9.7	45
Non exercise activity thermogenesis (kcal/kg/d)	24.6 ± 9.2	13.5–45.3	45
Body composition parameter			49
Fat mass (kg)	2.44 ± 4.4	−6.1–14.8	49
Total body water (L)	27.4 ± 2.9	19.4–33	49
Fat-free mass (kg)	37.4 ± 3.9	26.5–45.1	49
Extracellular mass (kg)	21.5 ± 4.3	5.9–34	49
Body cell mass (kg)	15.6 ± 3	8.4–21.4	49

Data are expressed as mean ± standard deviation. Abbreviations: Nnat, Neuronatin; BMI, body mass index.

**Table 3:** Comorbidities and Medication of Sample 2 (female patients with anorexia nervosa) and Sample 3 (female patients with obesity).

	Sample 2		Sample 3	
	*n* (% of Total)	Missing Data	*n* (% of Total)	Missing Data
Anorexia nervosa	49 (100%)	-	-	-
- restrictive type	25 (51%)	-	-	-
- purging type	12 (24%)	-	-	-
- atypical	12 (24%)	-	-	-
Obesity (BMI > 30 kg/m^2^)	-	-	79 (100%)	-
- Hyperphagic eating disorder	-	-	56 (71%)	-
- Binge eating disorder	-	-	17 (22%)	-
- Previous bariatric surgery	-	-	32 (40%)	-
Comorbidities				
Anxiety disorder	4 (8%)	1	6 (6%)	-
Somatoform disorder	4 (8%)	1	11 (14%)	3
Depression	24 (49%)	1	35 (44%)	-
Sleep-associated breathing disorder	0 (0%)	1	37 (47%)	-
Type 2 diabetes mellitus	0 (0%)	1	23 (29%)	-
Arterial hypertension	1 (2%)	1	48 (61%)	-
Hypercholesterinemia	23 (49%)	2	46 (58%)	-
Hypertriglyceridemia	2 (4%)	1	17 (22%)	-
Fatty liver disease	2 (4%)	1	51 (65%)	5
Medication				
Insulin	0 (0%)	1	6 (8%)	-
DDP-4 antagonists/ GLP-1 analogs	0 (0%)	1	4 (5%)	-
Other Antidiabetics	0 (0%)	1	15 (19%)	-
Steroids	2 (4%)	1	9 (11%)	-
Opioids	0 (0%)	1	5 (6%)	-
Antipsychotics	6 (12%)	1	12 (15%)	-
SSRI/SNRI	5 (10%)	1	20 (25%)	-
Tricyclic antidepressants	1 (2%)	1	9 (11%)	-
Other antidepressants	3 (6%)	1	2 (3%)	-
Tranquilizers, sedatives, hypnotics	0 (0%)	1	1 (1%)	-
Other psychopharmacological medication	1 (2%)	1	4 (5%)	-

Abbreviations: BMI, body mass index, DPP-4, dipeptidyl peptidase-4 inhibitor; GLP-1, glucagon-like peptide 1; SSRI, selective serotonin reuptake inhibitors; SNRI, serotonin-norepinephrine reuptake.

**Table 4:** General characteristics and psychometric variables of Sample 3 (female patients with obesity, *n* = 79).

General Parameter	Mean ± SD	Range	*n*
Plasma Nnat (pg/mL)	37.1 ± 27.7	0.2–111.9	79
Age (years)	44.8 ± 13.7	19–73	79
BMI (kg/m^2^)	48.8 ± 7.8	31.8–70.8	79
Psychometric Variables			
PHQ-9 Total	9.9 ± 6.1	0–25	78
GAD-7 Total	9.3 ± 5.7	0–21	79
EDI-2 Total	46.9 ± 12.6	20–79	75
- drive for thinness	32 ± 13.6	11–89	75
- bulimia	18.1 ± 10.6	0–71	75
- body dissatisfaction	55.9 ± 16.8	33–100	75
- ineffectiveness	32.6 ± 12.9	12–80	75
- perfectionism	21.6 ± 11.	8–60	75
- interpersonal distrust	25.4 ± 9.9	9–66	75
- interoceptive awareness	28.7 ± 9.2	8–55	75
- maturity fears	28.1 ± 12.1	11–88	75
PSQ-20 Total	56 ± 21.8	5–98	79
- worries	55 ± 27.4	0–100	79
- tension	59.4 ± 25.7	0–100	79
- joy	37.6 ± 25.3	0–100	79
- demands	46.6 ± 25.6	0–100	79

Data are expressed as mean ± standard deviation. Abbreviations: Nnat, Neuronatin; PHQ-9; Patient Health Questionnaire; GAD-7, Generalized Anxiety Disorder-7; EDI-2, Eating Disorder Inventory-2; PSQ-20, Perceived Stress Questionnaire.

## Data Availability

The data presented in this study are available on request from the corresponding author. The data are not publicly available due to data privacy.
